# The applications of single-cell multiomics in drug screening

**DOI:** 10.1016/j.pscia.2025.100090

**Published:** 2025-08-18

**Authors:** Qingming Xue, Hanyu Hu, Ruogu Wang, Fei Wu, Haiqing Xiong

**Affiliations:** a State Key Laboratory of Experimental Hematology, National Clinical Research Center for Blood Diseases, Haihe Laboratory of Cell Ecosystem, Institute of Hematology & Blood Diseases Hospital, Chinese Academy of Medical Sciences & Peking Union Medical College, Tianjin, 300020, China; b Tianjin Institutes of Health Science, Tianjin, 301600, China

**Keywords:** scMultiomics, Drug screening, Drug response, Drug resistance

## Abstract

Single-cell multiomics (scMultiomics) technologies and methods encompassing transcriptomics, genomics, epigenomics, proteomics, and metabolomics, together with associated computational tools have profoundly revolutionized disease research, enabling unprecedented dissection of cellular heterogeneity and dynamic biological responses. The use of scMultiomics technologies to study drug drug screening, actions and responses has not only unlocked novel avenues in precision drug screening but also transformed our understanding of how small molecules target specific cell types in cancer treatment, as well as their connections to disease etiology and progression from a high-resolution view of their functional diversity. In this review, we systematically explore how scMultiomics technologies develop and drive advancements in drug screening. With a specific focus on the applications in target identification, drug response, and drug resistance, we highlight how scMultiomics can link cellular-level insights with individualized drug screening, which in turn promises actionable strategies to improve therapeutic precision in drug development.

## Graphical abstract

**Figure:**
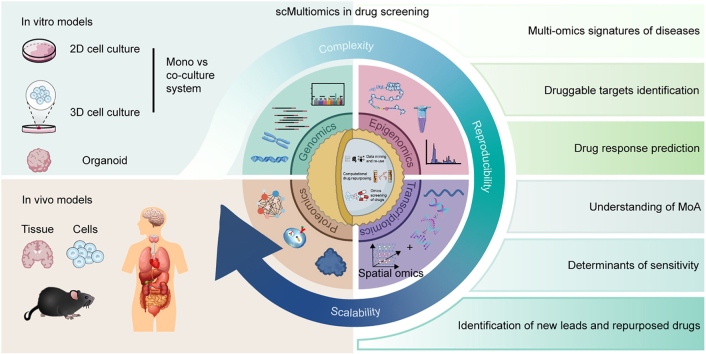

## 1 Introduction

Drug screening constitutes the central stage of modern pharmaceutical research and development, with the iterative advancements in its technological framework consistently pursuing two primary objectives: precise elucidation of molecular mechanisms underlying drug action [1,2] and enhancement of personalized prediction of therapeutic efficacy [2,3]. Target identification and mechanistic validation still prove to be non-trivial, although traditional target-/phenotype-based methods help address these challenges associated with drug screening.

Intratumoral and intertumoral heterogeneity, arising from a confluence of diverse factors, constitute fundamental hallmarks of cancer [[4], [5], [6]], critically driving therapeutic resistance and treatment failure [[7], [8], [9]]. While molecular and biochemical advances capture population-averaged tumor profiles [[10], [11], [12]], drug screening and discovery remain impeded by variable treatment sensitivity across cellular subpopulations. Traditional bulk sequencing cannot reflect their heterogeneity due to the ability of detecting an average level of molecular signals in tumor tissues. The advent of single-cell technologies in 2009 [13] has transformed biomedical research by enabling the profiling of individual cells, revealing previously obscured cellular diversity. Building upon this foundation, scMultiomics has emerged as a powerful approach that simultaneously profiles two or more molecular layers, such as the transcriptome, genome, epigenome, proteome, or even metabolome, within the same cell.

These advances have profound implications for drug screening and discovery, particularly in oncology. scMultiomics enables high-resolution mapping of drug responses, identification of rare resistant subpopulations, and mechanistic dissection of drug-gene interactions that are often overlooked by traditional bulk assays [[14], [15], [16], [17]]. It provides a framework to link molecular target engagement with downstream functional outcomes, accelerating the validation of therapeutic targets and biomarkers. Therefore, scMultiomics is also helping to identify appropriate preclinical disease models and offering new perspectives on drug mechanisms of action during clinical development.

In this review, we provide a comprehensive overview of recent scMultiomics innovations across the drug development continuum. We discuss their application in anticancer target identification, mechanistic insight into unexpected drug responses, and the design of precision medicine strategies. By highlighting how scMultiomics overcomes key limitations in conventional drug screening, such as the inability to resolve heterogeneous cell states or predict context-dependent drug effects, we aim to illustrate its potential to reshape future therapeutic development.

## 2 Traditional approaches in drug screening

**Fig. 1:**
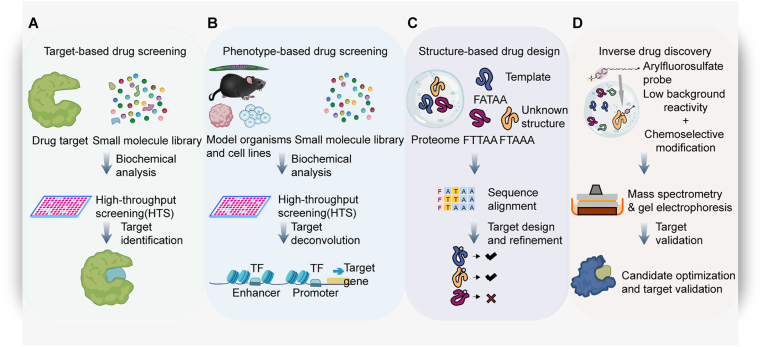
**Traditional approaches in drug screening**. (A) Target-based drug screening (TBDS): This approach begins with a specific drug target that may be tied to the pathophysiology of diseases. And a small-molecule library is then used to screen against the given target. The process includes biochemical analysis and high-throughput screening (HTS) to identify high affinity-binding molecules as lead compounds. (B) Phenotype-based drug screening (PBDS): It induces the required phenotypic changes in specific biological models and cell lines by screening a library of small molecule compounds. Following the initial screening, target deconvolution is essential to clarify the mechanisms of initial hits. (C) Structure-based drug design (SBDD): This method guides the design and optimization process by comparing a template structure with the structure of the unknown proteins to screen for structural models and pursue high-resolution refinement. (D) Inverse drug discovery (IDD): It utilizes arylfluorosulfate probes to conduct screening in selected biological cell lines to verify whether the target sites are related to the disease or outperforms current ones.

Broadly speaking, drug screening is the process of identifying and optimizing potential therapeutic compounds, culminating in the selection of a candidate drug for advancement to clinical trials. The program of drug screening aims to identify molecules capable of mitigating the effects of diseases [[18], [19], [20], [21]]. In general, traditional drug screening approaches fall broadly into four categories: (1) Target-based drug screening (TBDS); (2) Phenotype-based drug screening (PBDS); (3) Structure-based drug design (SBDD); (4) Inverse drug discovery (IDD) [22,23] (Fig. 1).

From the 1980s to the 2000s, the era of target-based drug screening witnessed significant advancements, with its theoretical underpinnings rooted in Emil Fischer's lock-and-key model. This model posits that drug molecules exhibit high specificity in binding to biological targets (e.g., receptors, enzymes), analogous to the complementary interaction between a key and a lock [24]. During this period, researchers employed in vitro assays to directly measure compound activity parameters, such as enzyme inhibition constants (Ki) and receptor binding affinities (Kd), to identify promising candidate molecules. The widespread adoption of high-throughput screening (HTS) technologies enabled the evaluation of tens of thousands of compound-target interactions per day. However, the clinical translation rate of small-molecule drugs derived from TBDS remains suboptimal, with only 9.4% achieving regulatory approval [24]. Phenotype-based drug screening (PBDS), another key approach, underwent a paradigm shift from a marginal approach to a mainstream strategy during 2000–2010. This target-agnostic screening modality evaluates compound efficacy by observing global phenotypic impacts on cellular proliferation, apoptosis, and migration, successfully overcoming reductionist limitations of target-centric approaches [25]. The core strength of phenotypic screening lies in preserving biological complexity, enabling therapeutic effects through polypharmacological interactions. This “black-box” approach successfully identified classical therapeutics like retinoic acid and rapamycin [26]. Both TBDS and PBDS employ large collections of structurally diverse small molecules. In contrast, the approach of structure-based drug design was driven by advancements in computational chemistry. Generative models demonstrate remarkable potential in dual-target inhibitor design, with experimental validation showing that computationally generated MEK1/mTOR dual-target inhibitors achieve >50% target protein activity inhibition at 1–10 ​μM concentrations [27]. Notably, mechanistic elucidation often required years of reverse pharmacological studies. This inverse drug discovery methodology surveys entire cellular proteome of a living cell against one small molecule reacting with Structurally distinct arylfluorosulfate, and therefore it facilitates the discovery of protein targets that are complementary to the combination of organic substructure and latent electrophile used [28].

Traditional drug screening approaches are fraught with limitations that contribute to the high attrition rate in drug development. Nearly 90% of drug candidates fail to achieve market approval due to insufficient efficacy, unforeseen adverse effects, and the high costs and time requirements of preclinical and clinical testing [29,30]. A key weakness lies in the inability to capture cellular heterogeneity and rare, yet crucial, cell populations that drive differential drug responses. These methods also fail to resolve complex, multilayered biological mechanisms and often overlook subtle but critical cellular reactions. These limitations hinder the exploration of disease-related mechanisms and the applications in target identification, drug response, dynamic modeling, and personalized prediction. More efforts are still warranted to refine the methods in drug screening.

## 3 Fundamentals of scMultiomics technology

### 3.1 The emergence of single-cell sequencing

The intrinsic limitations of conventional drug screening necessitate technological innovations to enhance resolution and throughput. The development of omics technology offers significant advantages in cost-effectiveness, high throughput, and automation. For example, DRUG-seq was developed for rapid and cost-effective drug screening [31]. Moreover, CP-seq advances this capability by identifying transcriptomic changes in individual cells following exposure to drug combinations and decoding corresponding drug barcodes. This integrated approach permits high-throughput, precise screening by directly associating specific combinatorial drug effects with transcriptional outcomes [32]. Collectively, these technological advancements bridge the gap between traditional screening and omics approaches, paving the way for advancements in single-cell resolution. However, conventional bulk omics technologies lack the resolution to discern cell-type-specific drug actions within complex tissue microenvironments, particularly for targeting highly heterogeneous pathogenic cell subsets in immune-mediated diseases [33,34]. Methodological and technological advances in single-cell sequencing have revolutionized the field of cancer biology and drug screening with an unprecedented level of granularity.

### 3.2 The development of the history and evolution of single-cell technologies

The first description of single-cell transcriptome analysis based on a next-generation sequencing platform was achieved in 2009 [13], which has unprecedentedly revealed dynamic variations in drug responses across the tumor microenvironment, immune cell subsets, and therapy-resistant clones in disease models [2,3,35,36]. The continuous evolution of single-cell technology, marked by enhanced throughput, improved accuracy, greater automation, and increased commercialization, has facilitated its broad application in resolving essential biological and clinical questions [37]. Furthermore, among different molecular modalities, transcriptomic analysis enables cell-type identification and provides a functional readout of global transcriptional activity. When integrated with epigenomic data, this approach can elucidate how various epigenetic mechanisms regulate genome architecture and gene-specific expression patterns. Concurrently, genomic information facilitates lineage reconstruction and helps evaluate how genetic modifications impact stem cell behavior, for example, by correlating specific mutations with dysregulated gene expression, functional impairments, and phenotypic alterations. Given these powerful applications, there is tremendous enthusiasm within the scientific community to develop scMultiomics technologies capable of simultaneous genome, epigenome, and transcriptome profiling in individual cells. Such integrated approaches promise to deliver unprecedented insights into cellular states and functions, potentially revolutionizing our understanding of biological systems and disease mechanisms.

### 3.3 The strategies of separating and detecting different layers in the same cells

ScMultiomics enables concurrent profiling of multiple molecular layers including genomic, epigenomic, transcriptomic, proteomic, and metabolomic modalities within individual cells. This integrated approach provides unprecedented resolution for deconvoluting gene regulatory networks, cellular heterogeneity, and physio-pathological mechanisms. Multiple strategies have been developed to isolate and detect molecular information of different layers in individual cells (Table S1). These strategies can be categorized into four groups: (1) Physical separation of different molecules. scTrio-seq uses micropipette manipulation to transfer the supernatant containing mRNA, enabling the physical separation of DNA methylome and mRNA [38]. (2) Biochemical separation. For example, G&T-seq adopts magnetic beads conjugated to biotin-labeled oligo-dT primers to capture mRNA, and the DNA is placed in the supernatant [39]. ChAIR also uses beads to pulldown the biotinylated double-stranded DNA [40]. (3) Information conversion. scNMT-seq uses a GpC methyltransferase to enzymatically label accessible chromatin, enabling the joint profiling of DNA methylation, chromatin accessibility, and the transcriptome following a bisulfite conversion step [41]. (4) Pre-library split. In ISSAAC-seq, researchers obtain the initial library through steps such as nuclei isolation and reverse transcription, after which the library is divided into two equal portions. One portion is applied to ATAC-seq and the other is applied to RNA-seq [42].

### 3.4 The throughput of single-cell experiments

**Fig. 2:**
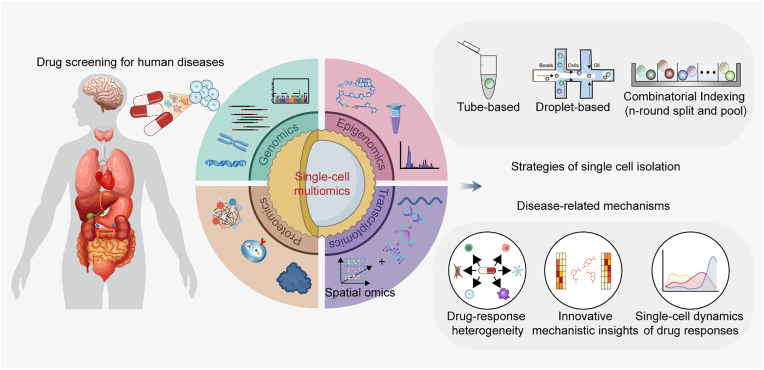
**Single-cell multiomics technology in drug screening for human diseases.** Overview and characteristics of single-cell multiomics in drug screening. Broadly defined, single-cell multiomics methods were developed for joint detection of multiple layers including genomics (mutations, copy number alterations), epigenomics (chromatin accessibility, DNA methylation, and histone marks), transcriptomics, and proteomics at single-cell resolution. Within the realm of single-cell multiomics strategies, the acquisition of single cells primarily relies on the utilization of the following two kinds of assays: low throughput (tube-based strategies) and high throughput (droplet-based and split-and-pool strategies). Single-cell technologies have revolutionized the field of target identification in anti-tumor drug discovery through providing insights into the molecular mechanisms underlying human diseases at single-cell level.

Currently, single-cell experiments broadly bifurcate into low-throughput and high-throughput platforms based on the experimental protocol and strategies. In classical plate-based or tube-based methods, cells are isolated via FACS, and library preparation is performed using manual or automated workflows. Molecular barcoding on plates allows manual handling of thousands of cells with limited read-out capabilities. The strategy based on microfluidic droplet and combinatorial Indexing (n-round split-and-pool) (applying iterative splitting and pooling protocols to enable scalable single-cell barcoding) mainly differs in how the cells are captured and pre-processed, which have massively increased the throughput of single-cell sequencing campaigns. Throughput can be further increased by super loading using hashing or in-situ barcoding (Fig. 2). For example, the scMultiomics platforms for joint detection chromatin accessibility and transcriptome are categorized by different barcoding strategies: Tube/plate-based low-throughput methods (scDam&T-seq, scCAT-seq), droplet-based high-throughput systems (ASTAR-seq, SNARE-seq), and combinatorial indexing approaches (Paired-seq, sci-CAR, SHARE-seq, ISSAAC-seq). Recently, a universal workflow named UDA-seq integrates a post-indexing step and systematically adapts existing droplet-based single-cell multimodal methods to enhance throughput [43]. These technologies permit quantitative inference of chromatin potential effects on transcription through enhancer regulatory modeling and cell-type-specific impact assessments on target genes.

### 3.5 The development of scMultiomics technology

**Fig. 3:**
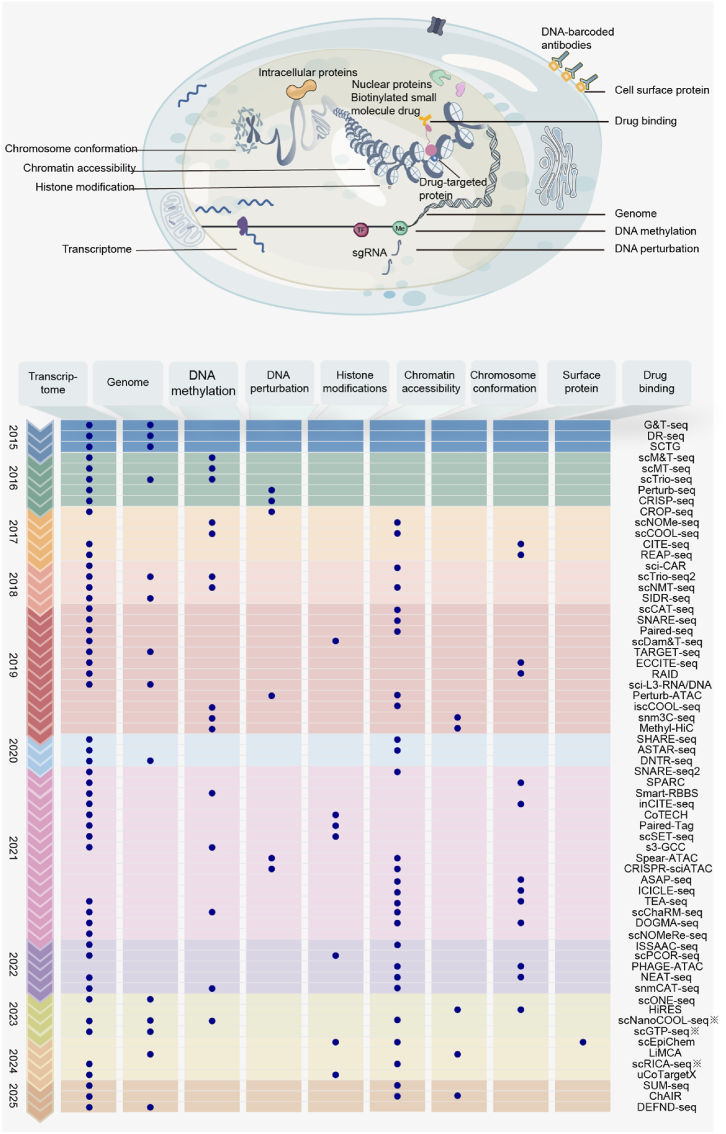
**Methods for single-cell multimodal omics analysis.** Schematic overview of molecular modalities captured in single-cell analysis (top), and a timeline of key technological developments enabling multimodal measurements (bottom). In addition to the genome, transcriptome, epigenome, and proteome, recent progress in using sc-multiomics (scEpiChem) to improve drug research by examining drug-chromatin interactions is also included. Representative technologies for each modality combination are summarized chronologically. ※: single-cell long-read sequencing technologies.

ScMultiomics is currently in a stage of rapid development with new technologies emerging one after another. To enhance scale and throughput, technologies like SUM-seq combine combinatorial fluidic indexing with single-plex assays, enabling the profiling of millions of cells across hundreds of samples [17]. In terms of expanding the classes of information, uCoTargetX enables joint analysis of multiple histone modifications and transcriptome in single cells by pre-assembled Ab-PAT-T7 complexes and biochemical separation strategy [44], which was developed based on single-cell ChIP-seq methods [[45], [46], [47], [48]]. SnmCAT-seq can achieve the joint profiling of transcriptome, DNA methylation, and chromatin accessibility [49]. Futhermore, to improve the applicability, scONE-seq achieves joint profiling of genome and transcriptome. Unlike other common scMultiomics technologies, it can be applied to the frozen biobanked samples because it could amplify the single-cell DNA and RNA without separating them [50]. Additionally, the emergence of single-cell long-read sequencing technologies enables full-length RNA and cDNA detection, not only addressing the mapping ambiguities associated with short-read platforms but also allowing precise reconstruction of transcript isoforms, detection of alternative splicing events, and accurate identification of gene fusion and structural variations at single-cell resolution. For example, scGTP-seq has been successfully applied to hepatocellular carcinoma (HCC) samples for the concurrent detection of genomic structural variants and extrachromosomal DNA along with transcriptomic features, offering valuable insights into tumor cell heterogeneity [51]. Overall, these newly developed technologies focus on expanding the detection capability of scMultiomics and enhancing its application value (Fig. 3).

Importantly, small molecules have close connections with intracellular signaling pathways and physiological changes of cells [52,53]. Some kinds of drugs interact with chromatin and influence gene expression [54,55]. Therefore, it is necessary to research the interaction mechanism between small molecules of drugs and chromatin. Some technologies such as Chem-map have made it possible to detect the interaction of drug and chromatin [56]. However, for an extended period of time, it was difficult to detect the drug-chromatin engagement at single-cell resolution. The recently developed scEpiChem technology directly addresses this gap. scEpiChem enables joint analysis of small molecule drug-target engagement and epigenome. It utilizes split-and-pool barcoding strategy which enabled detection of hundreds of thousands or even millions of single-cell profiles in an experiment. Furthermore, scEpiChem can distinguish between drug-target protein binding and drug-chromatin binding. This feature makes the scEpiChem more practical in the analysis of specific drug targets and related regulatory networks [57]. We contend that scEpiChem has great prospects for deciphering drug mechanisms, and more scMultiomics technologies that can identify small molecules-target binding will emerge in the future.

Looking forward, two key trends are shaping the future of single-cell analysis. The first is the integration of spatial context. Traditional scMultiomics technologies require tissue dissociation, which results in the loss of crucial spatial information. For example, SMA integrates histology, mass spectrometry imaging and spatial transcriptomics, enabling the simultaneous spatial profiling of metabolome and transcriptome [58]. Spatial-Mux-seq, a newly developed technology, achieves simultaneous profiling of two histone modifications, chromatin accessibility, transcriptome and proteins in a spatially resolved manner [59]. The development of spatial multiomics helps to reveal the spatiotemporal relationships between substances in different dimensions and can provide a more precise understanding of biological processes.

The second trend is the fusion of scMultiomics with functional genomics. The combination of scMultiomics and CRISPR links CRISPR-induced genetic perturbations to omic changes such as transcriptome change and epigenome change. Perturb-ATAC integrates CRISPR and ATAC-seq, it can analyze the influences of CRISPR-based perturbations on chromatin accessibility [60]. Perturb-FISH combines single-cell spatial transcriptomics and CRISPR perturbation, presenting intracellular effects of genetic perturbations more clearly and enhance the study of intracellular circuitry [61]. Overall, the combination of scMultiomics and CRISPR can deepen our understanding of cellular signaling regulatory network (Fig. 3).

## 4 Applications of scMultiomics in drug screening

### 4.1 The application of scMultiomics in detecting cell-type-specific drug response

#### 4.1.1 Transcriptomic-centered analysis of drug responses

**Fig. 4:**
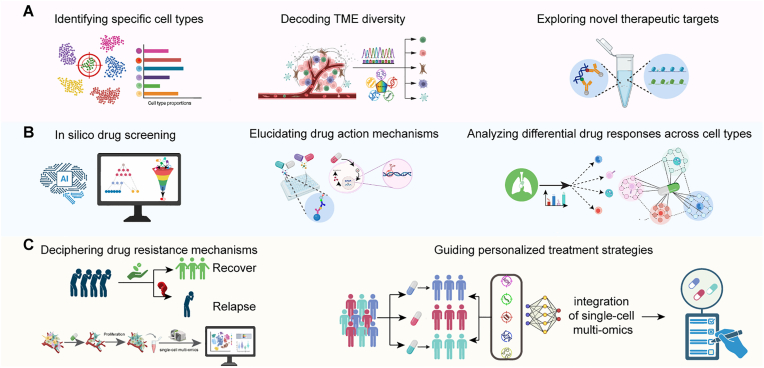
**The application scenarios of scMultiomics technologies in drug screening.** (A) ScMultiomics identifies cellular/TME heterogeneity in complex diseases for drug targets. (B) ScMultiomics deciphers the disease-related mechanisms based on the information of different modalities at the single-cell level. (C) ScMultiomics offers the chance of overcoming therapeutic resistance and advancing precision medicine.

Small molecule drugs mediate pharmacological effects through interactions with intracellular targets, modulating signaling pathway activation and epigenetic regulation [[62], [63], [64]]. The detection of cell-type-specific changes in response to drug treatment is a paramount objective for effective drug screening. Traditional bulk technologies generate population-averaged signals that obscure cellular heterogeneity and intra-population variations. In contrast, scMultiomics overcomes this limitation, offering a powerful new approach to drug screening by resolving cellular and TME heterogeneity in complex diseases (Fig. 4A, Table S2).

ScMultiomics has demonstrated remarkable capabilities in this context. For instance, the integration of multiplexed drug perturbation with single-cell RNA sequencing has successfully revealed distinct, cell-type-specific drug sensitivities and unexpected effects on the tumor microenvironment in glioblastoma [65]. Building on this, Tlemsani et al. integrated high-resolution methylome and transcriptome data with drug sensitivity analysis in small cell lung cancer (SCLC). This multiomics approach uncovered cell-type-specific responses to mTOR and AKT inhibitors across SCLC subtypes and elucidated the underlying molecular pathways [66]. Similarly, in acute myeloid leukemia, integrated platforms utilizing flow cytometry, DNA-seq, and RNA-seq have shown that drug responses differ markedly between cell subpopulations, highlighting the importance of cell-type-resolved analysis for both drug development and treatment decisions [67]. Drug2cell, a methodology for integrating spatial transcriptomics, single-cell RNA sequencing (scRNA-seq) and digital pharmacology, achieves precise spatial localization of identified pathogenic immune cells, pro-inflammatory T cell subsets (Th1/Th17 and Tc1/Tc17) in renal niches while simultaneously characterizing their phenotypes and functions. By constructing a “drug-gene-cell” interaction network from spatial and single-cell data [68]. The emergence of drug2cell and other burgeoning technologies transforms the concept of precisely mapping “drug-cell” interactions into a feasible reality***,*** establishing a new paradigm for cell-type-directed precision therapy.

#### 4.1.2 Proteomics-based analysis of drug responses

Beyond transcriptomics, advances in single-cell proteomics are enabling deep profiling of protein-level drug responses. Workflows are now capable of identifying cell-type-specific protein markers with high precision [69]. A specific application, single-cell chemical proteomics (SCCP), has been used to analyze A549 adenocarcinoma cells treated with methotrexate, camptothecin, and tomudex. This technique successfully identified early cellular subpopulations that were either committed or uncommitted to apoptosis, providing critical insights into cellular fate decisions post-treatment [70].

#### 4.1.3 AI-driven model in cell-specific drug responses

A primary application of AI is to predict cell-specific drug responses, bridging the gap between preclinical models and clinical samples (Fig. 4B). Frameworks like scIDUC integrate tumor single-cell transcriptomics with large-scale cell line drug screens to infer drug sensitivity at single-cell resolution [71]. The Scaden-CA model advances this by leveraging both transcriptomic and mutational data to deconvolute tumor composition and predict the drug sensitivity of each constituent cell type, thereby clarifying cell-type-specific mechanisms of action [72]. Critically, these predictive capabilities have shown clinical relevance. The PERCEPTION model, for example, demonstrates high accuracy in predicting therapeutic responses in multiple myeloma (AUC, 0.827) and breast cancer cohorts, and successfully identifies a TKI-resistant, MUC-high subpopulation in lung cancer, a finding corroborated by other AI-driven analyses linking AXL pathway activation to EGFR inhibitor resistance.

Collectively, these developments demonstrate that scMultiomics is transforming drug research. By enabling the precise detection and interpretation of cell-type-specific responses to pharmacological agents, these technologies are paving the way for the development of more targeted and effective therapies.

### 4.2 The application of scMultiomics in identifying drug targets

ScMultiomics is profoundly transforming drug target discovery by providing unprecedented resolution of cell-type-specific molecular features across transcriptional, epigenetic, and spatial landscapes. By integrating these diverse data layers, researchers can uncover novel, context-dependent therapeutic targets that are invisible to single-modality analyses.

#### 4.2.1 Joint profiling of gene expression and protein expression

A primary application lies in directly linking cell surface protein expression with transcriptomic states. Technologies such as CITE-seq (Cellular Indexing of Transcriptomes and Epitopes by Sequencing) are particularly powerful for identifying surface markers. For instance, in cancer research, integrating the expression of co-inhibitory/co-stimulatory receptors with the transcriptional profiles of T cells has enabled the discovery of novel immune checkpoint molecules, offering promising targets for immunotherapy [73]. Recent studies further exemplify this point: Single-cell transcriptomic analysis and ATAC-seq identified LILRB4 as highly enriched in pre-matured plasma cells of multiple myeloma patients versus those in durable remission, establishing it as a dual immunotherapy target for tumor cells and myeloid-derived suppressive cells [74]. These evidences efficiently pinpoint druggable surface proteins directly involved in cellular function and disease pathology.

#### 4.2.2 Joint profilings of gene expression and chromatin accessibility

Delving deeper into gene regulation, other multi-modal methods elucidate the interplay between the epigenome and transcriptome. Techniques like SNARE-seq (Simultaneous Nucleosome and RNA Expression sequencing) or the computational integration of scATAC-seq and scRNA-seq data can delineate how chromatin accessibility governs gene expression. This has been applied to map aberrant chromatin regions to oncogene expression in cancer cells, thereby identifying epigenetic regulators like histone deacetylases (HDACs) as actionable targets [75,76]. Besides, another case is that joint analyses of these modalities have uncovered aberrant transcriptional programming in therapy-resistant malignancies. For example, integrated scRNA-seq/scATAC-seq revealed enhanced transcriptional activation of primitive cells in pediatric AML relapse samples and nominated MEF2C as a therapeutic target [77]. Similarly, in the tumor microenvironment, these methods have revealed how immune cell-specific chromatin remodeling alters immune response genes, unmasking context-dependent checkpoint molecules as novel therapeutic targets [73,78].

#### 4.2.3 Joint profiling of gene expression and spatial information

Adding the crucial dimension of spatial context, spatial multiomics technologies map molecular profiles within the native tissue architecture. This is critical for understanding diseases driven by localized cellular interactions and microenvironments. In age-related macular degeneration (AMD), for example, spatial profiling uncovers cell-specific metabolic and transcriptional dysregulation in diseased retinal tissue, nominating pathways such as antioxidant signaling for therapeutic intervention [79]. scRNA-seq coupled with spatial transcriptomics in colorectal cancer exposed FAP ​+ ​fibroblasts/SPP1 + macrophage interactions as targetable barriers to immunotherapy [80]. The utility of scRNA-seq and spatial transcriptomics extend beyond human disease, for instance, in optimizing biomanufacturing by mapping the spatial expression of key enzymes (e.g., terpenoid synthases) to guide metabolic engineering in plants [81].

#### 4.2.4 Joint analysis of RNA splicing and other modality

Furthermore, scMultiomics can dissect post-transcriptional regulatory mechanisms, such as RNA processing. scRICA-seq (single-cell RNA isoform and chromatin accessibility sequencing) addresses this by co-assaying RNA splicing and chromatin state. In the context of neurodegeneration, this technology has linked splicing factor activity with epigenetic states in neural cells, identifying aberrant RNA splicing machinery as a potential therapeutic target for conditions like Alzheimer's disease [82]. Additionally, it has been reported that integrated analysis of depletion-like T cell states identified IL-15 as a potential immunotherapeutic target via multimodal characterization [83].

In summary, scMultiomics empowers drug target discovery by constructing integrated cellular state maps. By multidimensionally bridging molecular layers, from surface proteins and epigenome to RNA processing, within native spatial tissue architecture, these technologies drive the paradigm shift from generic target identification toward precision discovery of cell-type-resolved, context-dependent candidates for next-generation therapeutics.

### 4.3 The application of scMultiomics in drug resistance

Understanding therapeutic resistance at the single-cell level is crucial for designing durable therapies. While single-modality approaches like scRNA-seq have been invaluable, scMultiomics provides a far deeper, mechanistic understanding by simultaneously profiling multiple molecular layers within the same cell. This integrated approach moves beyond simply cataloging resistant cell states to uncovering the causal mechanisms driving them a feat often impossible with single-omics alone (Fig. 4C).

#### 4.3.1 Integration of transcriptome and epigenome

A key advantage of scMultiomics is its ability to directly link epigenetic landscapes to transcriptional outputs, thereby revealing the upstream regulatory logic of resistance. While scRNA-seq can identify which genes are upregulated in resistant cells, it cannot explain how they are activated. By integrating scATAC-seq with scRNA-seq, researchers can pinpoint the specific enhancers and promoters that become accessible to drive the expression of resistance genes. For example, in pediatric acute myeloid leukemia (pAML), the integration of single-cell epigenetic and transcriptomic data was crucial for tracing aberrant epigenetic kinetics from diagnosis to relapse, identifying key regulators that orchestrate cell cycle dysregulation and drive the emergence of resistant clones [77].

#### 4.3.2 Integration of transcriptome and proteome

Furthermore, scMultiomics is essential for resolving the frequent discordance between mRNA levels and functional protein expression. In refractory multiple myeloma, for example, longitudinal scRNA-seq identified peptidylprolyl isomerase A (PPIA) as a transcriptomic signature of resistance, but its functional validation as a therapeutic target requires confirmation at the protein level [84]. Technologies like CITE-seq directly address this gap. The functional genomics platform Perturb-CITE-Seq exemplifies this power by integrating CRISPR-Cas9 screening with simultaneous single-cell transcriptomic and proteomic analysis. This has not only validated known resistance pathways like IFNγ-JAK/STAT defects but also uncovered novel drivers, such as the loss of the protein CD58, which drives resistance independently of classical transcriptional pathways [85]. This ability to causally link a genetic perturbation to both RNA and protein consequences in one experiment provides unparalleled mechanistic clarity.

#### 4.3.3 Integration of transcriptome and lineage tracing

Finally, multiomics excels at connecting a cell's genotype to its dynamic resistant phenotype during clonal evolution. By combining lineage tracing or DNA sequencing with scRNA-seq, researchers can directly attribute a specific transcriptional program to a founding genomic event. For instance, in glioblastoma, this approach revealed that copy-number amplification of IRS1 and IRS2 in rare clones directly activated insulin/AKT signaling, allowing them to outcompete other cells under drug pressure [86]. This direct link from genotype to transcriptional phenotype is critical for understanding how pre-existing or acquired genetic alterations lead to functional resistance, providing a solid basis for designing rational combination therapies to preemptively target these evolutionary trajectories.

## 5 Conclusions and perspectives

ScMultiomics technology offers significant advantages for drug development. It elucidates cell subpopulations and molecular mechanisms difficult to detect with traditional methods. Although scMultiomics is still in its early stages, one of its key challenges lies in balancing data quality and throughput. Current high-throughput methods yield limited coverage of the epigenome and transcriptome at the single-cell level, making it difficult to distinguish true biological variation from technical noise. Integrating multi-omics data poses major challenges, including high dimensionality, heterogeneity across datasets, experimental inconsistencies, and the prevalence of missing values. As a result, many applications remain confined to proof-of-concept studies. Moving forward, more sensitive and efficient scMultiomics technologies will be essential to enable deeper biological insights and accelerate the discovery of better therapeutics. Future progress necessitates integrating multiomics and spatial data to comprehensively redefine tumor biology and drug action.

Clinically, scMultiomics can potentially improve therapeutic efficacy and survival outcomes by real-time monitoring of clinical trial patients (tracking resistance, clonal evolution, and biomarkers) [36]. By delineating tumor heterogeneity and resistance mechanisms, scMultiomics catalyzes development of targeted therapies against specific cell populations or pathways. Single-cell high-throughput screening accelerates drug discovery pipelines by shortening development cycles and reducing costs, while single-cell pharmacokinetic profiling optimizes lead compounds and boosts clinical success rates.

In conclusion, scMultiomics platforms fundamentally reshape tumor biology comprehension and hold transformative potential for oncology drug development. Continued technological refinement and integration with other high-throughput methodologies will pioneer deeper single-cell insights and accelerate targeted therapeutics. Notably, the fusion of scMultiomics and AI is creating a powerful virtuous cycle: high-resolution data fuels smarter models, which in turn generate testable hypotheses that guide more effective experiments. This synergy is fundamentally enhancing our ability to predict drug efficacy, understand resistance, and discover novel targets, accelerating the entire drug development pipeline toward a future of truly personalized medicine.

## CRediT authorship contribution statement

**Qingming Xue:** Writing – review & editing, Writing – original draft, Investigation, Data curation. **Hanyu Hu:** Writing – review & editing, Writing – original draft, Investigation, Data curation. **Ruogu Wang:** Writing – review & editing, Writing – original draft, Investigation, Data curation. **Fei Wu:** Writing – review & editing, Writing – original draft, Investigation, Data curation. **Haiqing Xiong:** Writing – review & editing, Writing – original draft, Funding acquisition, Conceptualization.

## Ethics approval

Not applicable.

## Declaration of generative AI in scientific writing

Not applicable.

## Funding information

H.X. was supported by grants from the Non-profit Central Research Institute Fund of Chinese Academy of Medical Sciences (2022-RC180-07); CAMS Innovation Fund for Medical Sciences (CIFMS) (2023-I2M-2–007, 2022-I2M-1–022); The National Natural Science Foundation of China (32470884); State Key Laboratory of Experimental Hematology Research Grant (Z22-09).

## Data availability

Not applicable.

## Declaration of competing interest

The authors declare that they have no known competing financial interests or personal relationships that could have appeared to influence the work reported in this paper.
